# Influence of HLA DRB1 alleles in the susceptibility of rheumatoid arthritis and the regulation of antibodies against citrullinated proteins and rheumatoid factor

**DOI:** 10.1186/ar2975

**Published:** 2010-04-06

**Authors:** Alejandro Balsa, Arancha Cabezón, Gisela Orozco, Tatiana Cobo, Eugenia Miranda-Carus, Miguel Ángel López-Nevot, José Luis Vicario, Emilio Martín-Mola, Javier Martín, Dora Pascual-Salcedo

**Affiliations:** 1 Servicio de Reumatología, Hospital Universitario La Paz, Paseo de la Castellana, 261, Madrid, 28046, Spain; 2 Sección de Inmunología, Hospital Universitario La Paz, Paseo de la Castellana, 261, Madrid, 28046, Spain; 3 Instituto de Parasitología y Biomedicina López-Neyra, Parque Tecnológico de Ciencias de la Salud, Avenida del Conocimiento, s/n, Armilla (Granada) 18100, Spain; 4 Unidad de Inmunología, Hospital Universitario Virgen de las Nieves, Avenida de las Fuerzas Armadas, 2, 18040 Granada, Spain; 5 Unidad de Histocompatibilidad, Centro de Transfusión de la Comunidad de Madrid, Avda. de la Democracia, s/n. 28032 Madrid, Spain

## Abstract

**Introduction:**

The purpose of this study was to investigate the association between HLA-DRB1 alleles with susceptibility to rheumatoid arthritis (RA) and production of antibodies against citrullinated proteins (ACPA) and rheumatoid factor (RF).

**Methods:**

We studied 408 patients (235 with RA, 173 non-RA) and 269 controls. ACPA, RF and HLA-DR typing were determined.

**Results:**

We found an increased frequency of HLA DRB1 alleles with the shared epitope (SE) in ACPA-positive RA. Inversely, HLA DRB1 alleles encoding DERAA sequences were more frequent in controls than in ACPA-positive RA, and a similar trend was found for HLA DR3. However, these results could not be confirmed after stratification for the presence of the SE, probably due to the relatively low number of patients. These data may suggest that the presence of these alleles may confer a protective role for ACPA-positive RA. In RA patients we observed association between SE alleles and ACPA titers in a dose-dependent effect. The presence of HLA DR3 or DERAA-encoding alleles was associated with markedly reduced ACPA levels. No association between RF titers and HLA DR3 or DERAA-encoding alleles was found.

**Conclusions:**

HLA DRB1 alleles with the SE are associated with production of ACPA. DERAA-encoding HLA-DR alleles and HLA DR3 may be protective for ACPA-positive RA.

## Introduction

Rheumatoid arthritis (RA) is a complex autoimmune disease that develops from the combined effects of genetic and environmental factors. It is estimated that the heritability of RA accounts for about 50% to 60%, and the most important genetic risk factors are the HLA class II molecules, which contribute to one third of the total genetic susceptibility [1,2]. There is extensive evidence for the association between certain HLA-DRB1 alleles with a conserved amino acid sequence (Q/RK/RRAA) at residues 70 to 74 in the third hypervariable region of the DRβ1 chain, the so-called shared epitope (SE), and susceptibility to and severity of RA [3,4].

Autoimmunity in RA is characterized by the presence of autoantibodies. Rheumatoid factor (RF) is not specific to RA as it may be present in other diseases and in healthy older individuals [5]. In contrast, anti-citrullinated protein antibodies (ACPAs) seem to play a pivotal role in the pathogenesis of RA as they are highly specific [6], can be detected years before the onset of symptoms [7,8], may predict progression to RA in patients with undifferentiated arthritis [9,10], are associated with the extent of joint destruction [11], and enhance disease severity in animal models of arthritis [12].

Recent studies, including our data, have demonstrated that SE alleles are associated only with ACPA-positive RA [13,14] and more strongly with ACPAs than with RA itself [15], suggesting that SE alleles may influence antigen presentation pathways leading to ACPA production; SE alleles have been used to subdivide patients into distinct immunopathogenetic disease classes [4]. Whereas the association between ACPA-positive RA and SE-containing HLA class II molecules is well established, the association between HLA-DR protective versus non-predisposing alleles and ACPA-negative RA is controversial. Certain HLA-DR alleles may reduce the risk of developing RA and have been termed 'protective alleles'; however, the definition of 'protective alleles' differs depending on the study [16], making all of these results difficult to interpret. Alleles with the DERAA motif at positions 70 to 74 in the third hypervariable region have been associated with a reduced risk of RA susceptibility [17,18] and less severe disease [17,19,20], whereas other studies have found conflicting results regarding HLA-DR3 [21-24].

Reproducing genetic associations is very important, and studies performed in cohorts outside of North America or Northwest Europe are especially welcome due to differences in allele frequencies and genetic background. To better understand the effect of HLA in RA in the Spanish population, we investigated the association of SE-containing HLA-DRB1 alleles with susceptibility to RA and then examined the possible protective effect of HLA-DR3 and DERAA-encoding alleles. Finally, the effects of these alleles on the magnitudes of RF and ACPA production were determined.

## Materials and methods

We studied 408 patients referred to the early arthritis clinic of La Paz University Hospital, Madrid. Data from this cohort have been previously reported [14]. At enrollment or during follow-up, 235 patients fulfilled the 1987 American College of Rheumatology criteria for RA and 173 were diagnosed with non-RA (mainly undifferentiated arthritis, psoriatic arthritis, reactive arthritis, and other connective tissue diseases) (Table 1). Most of the patients (91%) in the two groups have been followed up with for more than 1 year (mean follow-up of 6.5 years). We included 269 healthy volunteers as controls. The study was approved by the La Paz University Hospital ethics committee, and all subjects were of Spanish origin and provided written informed consent.

**Table 1:** Baseline characteristics of the patients in the study

	RA (n = 253)	Non-RA (n = 173)
Age in years, mean ± standard deviation	62 ± 12	50 ± 14
Percentage of patients who are women	64	55
ACPA-positive, number (percentage)	143 (61.9%)	9 (5.6%)
RF-positive, number (percentage)	169 (72.5%)	27 (15.9%)
Duration of symptoms in weeks, median (range)	20 (3-52)	15 (2-52)

Percentages exclude missing data.

ACPA, anti-citrullinated protein antibody; Non-RA, other arthropathies; RA, rheumatoid arthritis; RF, IgM rheumatoid factor.

For every patient, laboratory tests were performed on blood samples that were obtained during the patient's first visit to the clinic (before treatment with disease-modifying antirheumatic drugs) and that were stored at -40°C. RF was measured by nephelometry (Behring Nephelometer Analyzer II; Dade Behring, Inc., Deerfield, IL, USA) with a detection limit of 15 U/mL, and ACPAs were determined by a second-generation anti-CCP-2 antibody enzyme-linked immunosorbent assay (ELISA) (Immunoscan RA Mark 2; Euro-Diagnostica, Arhem, The Netherlands) with a cutoff level of 25 arbitrary units per milliliter in accordance with the instructions of the manufacturer. The range of measurement was 0 to 1,600 U/mL, and all values higher than this upper limit were truncated and considered to be 1,600 U/mL for the analysis.

In DNA samples obtained from peripheral blood of controls and cases, HLA class II alleles were genotyped using a reverse dot-blot kit with sequence-specific oligonucleotide (SSO) probes (Dynal RELITM SSO HLA-DRB1 typing kit; Dynal Biotech, Bromborough, UK). When necessary, high-resolution typing of HLA-DRB1*03 samples was performed using Dynal AllSetTM SSP DRB1*03. The following alleles were considered SE-positive: DRB1*0101, *0102, *0401, *0404, *0405, *0408, *0410, *1001, and *1402. DERAA-encoding alleles were HLA-DRB1*0103, *0402, *1102, *1103, *1301, *1302, and *1304.

Statistical analysis was performed using the Statistical Package for the Social Sciences, version 10.0 (SPSS Inc., Chicago, IL, USA). Odds ratios (ORs) and proportions were compared by the chi-square test. Differences in values between groups were analyzed using Kruskal-Wallis and Mann-Whitney *U *tests for non-parametric data.

## Results

Of the 253 patients with RA, 143 (61.9%) had ACPAs and 169 (72.5%) RF (Table 1). The presence of both autoantibodies was strongly associated with RA (OR 27.4, 95% confidence interval [CI] 14.08 to 53.34, *P *< 0.001 and OR 14.99, 95% CI 9.18 to 24.45, *P *< 0.001, respectively). In non-RA patients, only 9 (5.6%) and 27 (15.9%) had ACPAs and RF, respectively (Table 1). Double-positivity for both ACPAs and RF was found in 137 (59.3%) patients with RA and in 6 (3.5%) patients with other arthropathies.

The distribution of our study population with respect to HLA-DRB1 genotypes and alleles is shown in Tables 2 and 3. As described previously, an increased frequency of HLA-DRB1 alleles encoding the SE was found in patients with RA compared with non-RA and control subjects (56.2% versus 35.5%, OR 2.33, 95% CI 1.56 to 3.5, *P *< 0.001 and 56.2% versus 41.6%, OR 1.8, 95% CI 1.26 to 2.56, *P *= 0.001, respectively). There was a dose-dependent effect of SE-encoding HLA-DRB1 alleles: 29.8% of RA cases and 24.9% of controls had one allele (OR 1.89, 95% CI 1.13 to 3.13), whereas 14% of RA cases and 5.6% of controls were homozygous for SE-encoding HLA-DRB1 alleles (OR 3.97, 95% CI 1.93 to 8.16) (Table 3).

**Table 2:** Distribution of HLA-DRB1 alleles in the study population

		RA (n = 235)	Non-RA (n = 173)	Controls (n = 269)
SE	*0101	49 (10.4%)	15 (4.3%)	32 (5.9%)
	*0102	17 (3.6%)	13 (3.7)	16 (3.0%)
	*0401	27 (5.7%)	10 (2.9%)	23 (4.3%)
	*0404	19 (4%)	7 (2.0%)	10 (1.8%)
	*0405	27 (5.7%)	9 (2.6%)	17 (3.1%)
	*0408	4 (0.8%)	5 (1.4%)	8 (1.5%)
	*1001	18 (3.8%)	7 (2.0%)	18 (3.3%)
	*1402	4 (0.8%)	2 (0.6%)	3 (0.5%)
DR3	*03	43 (9.1%)	54 (15.6%)	55 (10.2%)
DERAA	*0103	4 (0.8%)	9 (2.6%)	14 (2.6%)
	*0402	7 (1.5%)	6 (1.7%)	10 (1.8%)
	*1102	6 (1.3%)	5 (1.4%)	11 (2.0%)
	*1103	7 (1.5%)	7 (2.0%)	12 (2.3%)
	*1301	25 (5.3%)	20 (5.8%)	27 (5.0%)
	*1302	11 (2.3%)	1 (0.3%)	5 (0.9%)
	*1304	-	-	-

Percentages relate to number of total alleles.

Data are presented as number and percentage of patients in each group. DERAA, HLA with DERAA-encoding alleles; DR3, HLA-DR3; Non-RA, other arthropathies; RA, rheumatoid arthritis; SE, shared epitope alleles.

**Table 3:** Distribution of HLA-DRB1 genotypes

	RA (n = 235)		Non-RA (n = 173)		Controls (n = 269)	
			
	Number	Percentage	Number	Percentage	Number	Percentage
SE/SE	33	14.0	7	4.0	15	5.6
SE/-	70	29.8	31	17.9	67	24.9
SE/DR3	15	6.4	12	6.9	16	5.9
DR3/-	21	8.9	25	14.4	23	8.6
DR3/DR3	-	-	5	2.9	2	0.7
SE/DERAA	14	6.0	11	6.3	14	5.2
DERAA/-	29	12.3	26	15.0	39	14.5
DERAA/DERAA	5	2.1	2	1.1	7	2.6
DR3/DERAA	7	3.0	7	4.0	12	4.5
No alleles (-/-)	41	17.4	47	27.1	74	27.5

DERAA, HLA with DERAA-encoding alleles; DR3, HLA-DR3; Non-RA, other arthropathies; RA, rheumatoid arthritis; SE, shared epitope alleles.

The frequency of HLA-DR3 alleles was lower in the RA group than in the non-RA group (18.3% versus 28.3%, OR 0.57, 95% CI 0.36 to 0.90, *P *= 0.012) but was similar to that of the healthy controls (18.3% and 19.7%, respectively). No differences in the DERAA-encoding allele distribution were found among the three groups (RA 23.4%, non-RA 26.6%, and controls 26.8%, *P *= 0.39). Next, we studied the effect of the presence of HLA-DR3 or DERAA-encoding alleles in combination with the SE on susceptibility to RA. In the presence of one SE allele, an HLA-DR3 or DERAA-encoding allele reduced the risk of developing RA, although the effect was not statically significant (OR 1.11, 95% CI 0.51 to 2.43 for HLA-DR3 and OR 1.04, 95% CI 0.46 to 2.36 for DERAA-encoding alleles) (Table 3). Finally, to study the effect of the presence of HLA-DR3 or DERAA-encoding alleles on susceptibility, we combined homozygous and heterozygous patients and controls for these alleles and compared them with 'neutral' alleles. No differences in susceptibility were found for HLA-DR3 alleles (OR 1.53, 95% CI 0.76 to 3.04) or DERAA-encoding alleles (OR 1.33, 95% CI 0.74 to 2.39).

To investigate whether HLA-DR alleles were associated with different RA phenotypes, we divided our RA population according to ACPA status (Table 4). We found a higher frequency of SE alleles in ACPA-positive RA compared with healthy controls (67.8% versus 41.6%, OR 2.95, CI 1.93 to 4.53, *P *< 0.001), but we did not observe this relationship with ACPA-negative RA patients and controls (35.2% versus 41.6%, *P *= 0.31), indicating that SE alleles are associated with ACPA production in RA. Inversely, HLA-DRB1 alleles encoding the DERAA sequence were more frequent in controls than in ACPA-positive RA patients (26.8% versus 17.5%, OR 0.58, CI 0.34 to 0.96, *P *= 0.03), and a similar trend was found for HLA-DR3 (19.7% versus 12.6%, OR 0.32 to 1.04, *P *= 0.07). However, the frequencies of HLA-DR3 were similar in controls and ACPA-negative RA patients, indicating that the presence of these alleles confers a protective role for ACPA-positive RA only. To further investigate whether the decreased frequencies of DERAA and HLA-DR3 alleles were secondary to the increased frequencies of SE in ACPA-positive RA patients, we stratified our study population by the presence of SE. A trend was observed for HLA-DR3 only, and no effect was observed for DERAA-encoding alleles, probably because of the low numbers of patients remaining (Table 5). No effect on RF of HLA-DR3 alleles (OR 1.10, 95% CI 0.43 to 2.83, *P *= 0.8) or DERAA-encoding alleles (OR 0.78, 95% CI 0.28 to 2.15, *P *= 0.5) was observed.

**Table 4:** Association of HLA-DRB1 alleles with the SE, HLA-DR3, and DERAA motif with ACPA^+ ^or ACPA^- ^rheumatoid arthritis

		Controls (n = 269)	Rheumatoid arthritis patients (n = 231)					
			
			**ACPA**^**+**^**(n = 143)**			**ACPA**^**-**^**(n = 88)**		
				
				*P *value	OR, 95% CI		*P *value	OR, 95% CI
SE	Absence, n (%)	157 (58.4)	46 (32.2)	0.0001	2.95, 1.93-4.53	57 (64.8)	0.31	0.76, 0.46-1.27
	Presence, n (%)	112 (41.6)	97 (67.8)			31 (35.2)		
DR3	Absence, n (%)	216 (80.3)	125 (87.4)	0.07	0.58, 0.32-1.04	63 (71.6)	0.10	1.61, 0.93-2.80
	Presence, n (%)	53 (19.7)	18 (12.6)			25 (28.4)		
DERAA	Absence, n (%)	197 (73.2)	118 (82.5)	0.03	0.58, 0.34-0.64	58 (65.9)	0.22	1.41, 0.84-2.37
	Presence, n (%)	72 (26.8)	25 (17.5)			30 (34.1)		

ACPA, anti-citrullinated protein antibody; CI, confidence interval; DERAA, DERAA-encoding allele; DR3, HLA-DR3; OR, odds ratio; SE, shared epitope allele.

**Table 5:** Association of HLA-DR3 and DERAA-encoding alleles stratified by the presence of shared epitope alleles with ACPA^+ ^rheumatoid arthritis

		Controls (n = 269)	ACPA-positive RA patients (n = 143)		
			
				*P *value	OR (95% CI)
SE-positive	DR3-positive	16 (14.3)	8 (8.2)	0.17	0.53 (0.22-1.22)
	DR3-negative	96 (85.7)	89 (91.8)		
SE-negative	DR3-positive	37 (23.6)	10 (21.7)	0.84	0.90 (0.40-1.98)
	DR3-negative	120 (76.4)	36 (78.3)		
SE-positive	DERAA-positive	14 (12.5)	10 (10.3)	0.85	0.58 (0.34-1.90)
	DERAA-negative	98 (87.5)	87 (89.7)		
SE-negative	DERAA-positive	58 (36.9)	15 (32.6)	0.72	0.82 (0.41-1.65)
	DERAA-negative	99 (63.1)	31 (67.4)		

Percentages relate to total SE-positive or SE-negative controls.

ACPA, anti-citrullinated protein antibody; CI, confidence interval; DERAA, DERAA-encoding allele; DR3, HLA-DR3; OR, odds ratio; RA, rheumatoid arthritis; SE, shared epitope allele.

**Figure 1:**
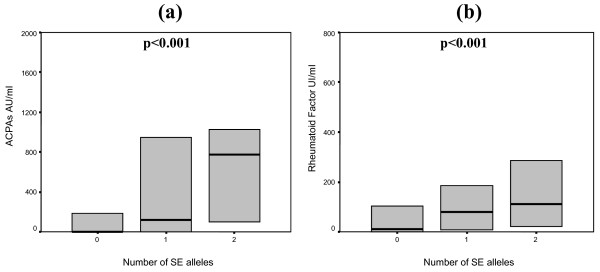
**Box plots of anti-citrullinated protein antibody (ACPA) levels (a) and rheumatoid factor (b) in rheumatoid arthritis patients depending on the number of shared epitope (SE) alleles**. ACPA levels are expressed as arbitrary units per milliliter (AU/mL), and rheumatoid factor levels are expressed as international units per milliliter (UI/mL). Each box represents the 25th to 75th percentiles. Lines outside the boxes represent the 10th and 90th percentiles, and lines inside the boxes represent medians. Differences in values between groups were analyzed using the Kruskal-Wallis test for non-parametric data.

In patients with RA, we observed a dose-dependent association between the presence of SE alleles and titers of RF and ACPAs (Figure 1). With two SE alleles, ACPA had a median value of 915 arbitrary units per milliliter in our ELISA (with an interquartile range [IQR] of 124 to 1,500). With one SE allele, there was a median ACPA of 400 (0 to 1,050), and with no alleles, it was 0 (0 to 650) (*P *< 0.001). The presence of HLA-DR3 or DERAA-encoding alleles was associated with markedly reduced ACPA levels. In the presence of HLA-DR3, ACPA had a median value of 0 (0 to 594), whereas in the absence of HLA-DR3, ACPA had a median value of 360 (0 to 1,000) (*P *= 0.002). ACPA was 0 (0 to 653) in the presence of DERAA-encoding alleles versus 310 (0 to 1,000) in the absence of any DERAA-encoding alleles (*P *= 0.013). No association between RF titers and HLA-DR3 or DERAA-encoding alleles was found.

**Figure 2:**
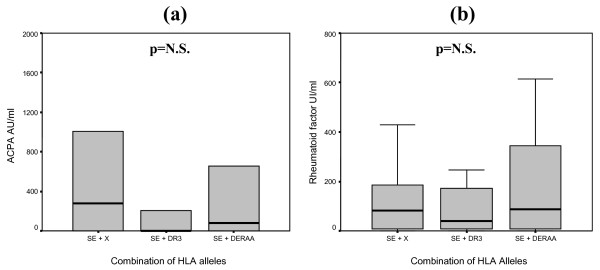
**Box plots of anti-citrullinated protein antibody (ACPA) levels (a) and rheumatoid factor (b) in rheumatoid arthritis patients depending on the presence of one shared epitope allele (SE) combined with an HLA-DR3 or DERAA-encoding allele**. ACPA levels are expressed as arbitrary units per milliliter (AU/mL), and rheumatoid factor levels are expressed as international units per milliliter (UI/mL). Each box represents the 25th to 75th percentiles. Lines outside the boxes represent the 10th and 90th percentiles, and lines inside the boxes represent medians. Differences in values between groups were analyzed using Kruskal-Wallis test for non-parametric data. N.S., not significant.

Next, we investigated the influence of DERAA-encoding alleles or HLA-DR3 together with one SE allele on RF and ACPA production. Although ACPA levels were lower in the presence of HLA-DR3 or DERAA alleles in combination with one SE allele (SE/DR3 or SE/DERAA) than in HLA-DR SE/- patients, these differences were not statistically significant: median 500 (IQR (34 to 1,250) in SE/- versus 468 (0 to 760) in SE/DERAA and 32 (0 to 900) in SE/DR3 (*P *= 0.11). No similar trend for RF was found (Figure 2).

## Discussion

Since 1987, when Gregersen and colleagues [25] formulated the SE hypothesis, several authors have investigated the relationship between RA and SE-encoding HLA-DRB1 alleles. An important discovery is that these alleles are associated with ACPAs in RA irrespectively of RF status [26]. Moreover, it was recently demonstrated that SE alleles are not associated with RA but are specific for a disease characterized by ACPAs [15]. Our results confirm these later observations as in our cohort ACPA-positive RA was strongly associated with the SE, with a gene-dose effect.

It was proposed that SE alleles function as immune response genes in the development of ACPAs as no differences in ACPA levels were found between RA patients with one or two SE copies [15]. In our cohort, as in other studies, a risk hierarchy in ACPA production with a significant dose effect in patients with two predisposing alleles was found [23]. Some SE alleles frequent in Northern Europe, such as DRB1*0401 and *0404, appear to confer a much greater degree of risk for RA [27-29] and are associated with higher levels of ACPAs than the DR1 or DR10 alleles are [28,29]. We have reported that, in our Spanish inception cohort, DR1 and DR4 alleles with the SE are equally represented [30] and that DR10 is a frequent SE allele in the Spanish population [31]. Therefore, it is possible that DR1- or DR10-heterozygous patients or both have lower levels of ACPAs than DR4 patients and that DR1- or DR10-homozygous patients or both have higher levels of ACPAs.

A lack of association between ACPAs and SE alleles, when they were analyzed together, has been described, with 68% of SE-negative patients having ACPAs compared with 32% in our cohort [29]. One reason for the lack of association may be the influence of external risk factors. Tobacco exposure is a well-known environmental risk factor for the development of ACPAs in the presence of SE alleles [32] but also in their absence [33]. Increased ACPA levels in tobacco users have been reported, but these effects were relevant for DR1 and DR10 alleles only, with no significant effect on DR4 patients who had higher levels regardless of tobacco exposure [28]. Other reasons may be that not only inherited SE alleles but also non-inherited SE alleles from the mother (NIMA) can be associated with RA susceptibility [34], and SE-positive cells persist in SE-negative women as a consequence of pregnancy (microchimerism) [35].

There are no well-established genetic risk factors for ACPA-negative RA [4]. Two studies have reported an increased risk of ACPA-negative RA associated with the presence of HLA-DR3 [21,22]; however, our results as well as those of two independent groups [23,24] do not support that finding. Instead, in HLA-DR3 carriers, we have found a reduced risk of ACPA-positive RA only. Another interesting finding of our study is that, as reported previously [21], HLA-DR3 carriers had lower levels of ACPAs compared with DR3 non-carriers. As this result may have been due to the reduced risk of ACPA production, we further analyzed the effect of HLA-DR3 in the presence of one SE allele and found a reduction in ACPA levels. The fact that we did not observe levels above the upper limit of detection of our ELISA may limit our capacity to demonstrate statistically significant differences.

The higher frequency of HLA-DR3 found in non-RA patients in our cohort was not unexpected. HLA-DR3 has been associated with several autoimmune diseases and with autoantibody production, mainly as part of an extended haplotype designated A1;B8;DR3 [36,37]. Whether the association with autoimmune diseases is caused by HLA-DR3 itself or by other nearby genes in linkage disequilibrium remains to be determined [21].

It has been proposed that some HLA class II alleles with a neutral or negative electric charge in their P4 pocket, such as DERAA-encoding alleles, reduce the risk of developing RA [17,23,38]; however, while the RA SE is consistently and reproducibly associated with RA in Caucasians, the 'protective' effect of some HLA alleles is far from fully accepted [16]. Our results are in accord with those of recent reports [27] and do not confirm that hypothesis. As with HLA-DR3, HLA DERAA-encoding alleles were associated with a reduced risk of ACPA-positive RA only, but this effect disappeared after stratification for the presence of the SE. As these same alleles have been associated with a reduced risk of RA [17,18], it is possible that these results are due to the low number of patients remaining after stratification, and new studies including greater numbers of patients are needed to clarify this effect.

HLA-DR3 and HLA DERAA-encoding alleles were associated with lower levels of ACPAs, and this finding may explain the association with less severe disease [19,20]. The progression of joint damage has been related to levels of ACPAs, both in RA patients [39,40] and in animal models of arthritis [12], so the previously described less severe disease in DERAA allele carriers may be due to lower levels of ACPAs. The reason for the reduced levels of ACPAs in the presence of HLA-DR3 or DERAA-encoding alleles is not clear, but it has been suggested that some non-associated MHC (major histocompatibility complex) class II molecules may contain P4 pockets that lack the proper size or charge to effectively accommodate the large polar side chains of citrulline, and they would be unable to bind to and present modified citrullinated peptides [41,42].

Overall, our data confirm the considerable complexity of HLA class II associations with RA. Many of the previously reported inconsistencies may be due not only to genetic differences or the influence of other genes in linkage disequilibrium but to the interaction with environmental risk factors. In summary, in a Spanish population, this study confirmed the previously reported observation that ACPA-positive RA is strongly associated with the HLA with the SE, with a gene-dose effect. However, our results do not support the hypothesis that HLA-DR3 or DERAA-encoding alleles protect against RA or are associated with ACPA-negative RA but rather that they only reduce the risk of ACPA-positive RA.

## Conclusions

In this study, we have examined the relationship between HLA-DRB1 alleles and RA in an inception cohort of early arthritis patients, and we confirm previous evidence of an association between SE-containing HLA-DRB1 alleles and ACPA-positive RA. Our results do not support a protective effect of HLA-DR3 or DERAA-encoding alleles against RA, nor are these alleles associated with ACPA-negative RA in the Spanish population. Instead, we suggest that these alleles reduce the risk of ACPA-positive RA, but these results must be confirmed with a larger number of patients or by analyzing pooled data from reported cohorts. The presence of these alleles was associated with lower levels of ACPAs, but in the presence of one SE allele, this interaction could not be demonstrated.

## Abbreviations

ACPA: anti-citrullinated protein antibody; CI: confidence interval; ELISA: enzyme-linked immunosorbent assay; IQR: interquartile range; OR: odds ratio; RA: rheumatoid arthritis; RF: rheumatoid factor; SE: shared epitope; SSO: sequence-specific oligonucleotide.

## Competing interests

The authors declare that they have no competing interests.

## Authors' contributions

AB, AC, JM, EM-M and DP-S participated in the design of the study, helped in the statistical analysis, and helped draft the manuscript. TC and EM-C participated in data collection and helped draft the manuscript. GO helped in the statistical analysis and participated in the design of the study. MAL-N and JLV carried out the HLA determination, analyzed and interpreted the data, and helped in the manuscript preparation. All authors read and approved the final manuscript.
